# Incisional negative pressure wound therapy for the prevention of surgical site infection after open lower limb revascularization – Rationale and design of a multi-center randomized controlled trial

**DOI:** 10.1016/j.conctc.2019.100469

**Published:** 2019-10-14

**Authors:** Francis Rezk, Håkan Åstrand, Stefan Acosta

**Affiliations:** a Department of Clinical Sciences, Malmö, Lund University, Sweden; b Vascular Center, Department of Cardiothoracic and Vascular Surgery, Skåne University Hospital, Malmö, Sweden; c Department of Surgery, Jönköping Hospital, Jönköping, Sweden

**Keywords:** Surgical site infection, Negative pressure wound therapy, Lower limb revascularization, Thrombendarterectomy, Bypass, Study protocol

## Abstract

**Introduction:**

Lower limb revascularization with inguinal incisions is a common vascular surgical procedure. Due to risk of injury to lymphatic vessels and a diverse bacterial flora in the groin, surgical site infections (SSI) represent a common and sometimes life-threatening complication. While transverse incisions in endovascular aneurysm repair has a low SSI rate, vertical incisions in thrombendarterectomy (TEA) has a higher risk and bypass the highest risk. This randomized controlled trial (RCT) will investigate the protective role of negative pressure wound therapy (NPWT) on closed inguinal incisions in elective vascular surgery undergoing TEA and bypass procedures, respectively, to prevent SSI.

**Methods:**

This RCT registered at ClinicalTrials.gov (Identifier: NCT01913132) compares the effects of a NPWT dressing (PICO™, Smith & Nephew, UK) to standard wound dressing on postoperative SSI. The multi-center study includes two distinct vascular procedures with different SSI risk profiles: TEA and lower limb bypass. Three hundred and fifty-eight groin incisions are anticipated to be included in the TEA group and 133 inguinal incisions in the bypass group. Bilateral inguinal incisions will be randomized to NPWT in one groin and control dressing in the contralateral groin, and this dependency was accounted for in sample size calculation and will be addressed in data analysis.

**Discussion:**

This RCT attempts to evaluate the potential benefit of NPWT on closed inguinal incisions after two distinct vascular procedures at high risk of SSI. Outcome of this trial could have implications on postoperative wound care in both vascular and non-vascular surgical patients.

## 1 Introduction

Lower limb revascularization relies on access to the common femoral artery. The inguinal region contains, however, a high concentration of virulent bacteria [1] due to its close proximity to the perianal region and open lower limb revascularization is associated with increased risk of surgical site infections (SSI). Meticulous surgical technique during open vascular surgery is necessary to minimize the risk of postoperative lymphatic wound complications [2]. Both patient- and procedure-related factors contributes to the risk for surgical site infections (SSI) [3]. It appears that extension of incisions are associated with increased risk of SSI during open vascular procedure [4]. Prospective series on open lower limb revascularization is not seldom associated with SSI rates of more than 20% [5]. Apart from being common, consequences may be devastating as severe deep SSI may lead to severe bleeding complications and/or limb loss and/or death.

Negative pressure wound therapy (NPWT) on closed, clean incisions, has emerged as a prophylactic option to reduce the risk of SSI in high-risk incisions [[6], [7], [8]]. A recent meta-analysis on randomized control trials (RCT) compared NPWT to standard dressing for closed groin incisions after vascular surgery and found a reduced SSI rate. This meta-analysis relied on the use of Prevena™ NPWT system. No RCT has been published using the alternative PICO™ device on vascular surgical incisions. In contrast to Prevena™, PICO™ is a canisterless, disposable NPWT system, where the dressing pad works mainly by evaporation of fluid [9]. NPWT decreases hematoma and seroma formation mainly by enhancing the endogenous draining capacity of the lymphatic system [10]. NPWT induces less biomechanical stress around skin and subcutaneous suture lines [11], which probably facilitate tissue apposition creating a better microbial barrier and decrease scar formation. There appear to be little perfusion changes around the wound edges measured by laser Doppler velocimetry before and after application of PICO™ on incisional wounds [12].

Patients undergoing inguinal vascular surgical incisions have very different SSI rate due to indication of surgery and procedure. While patients undergoing elective endovascular aneurysm repair (EVAR) have a low inguinal SSI rate of a few percent, those undergoing elective open lower limb revascularization have SSI rates around 30% [13]. Based on these different SSI rates, two sample size calculations were conducted and two separate RCTs launched [14]. However, patients undergoing elective open lower limb revascularization is very heterogenous in terms of SSI risk. Those undergoing lower limb bypass surgery has double as high SSI risk as the group undergoing local thrombendarerectomy (TEA) [15]. Therefore, a study protocol for a multi-centre RCT on incisional NPWT compared to standard dressing in two subgroups, local TEA group and bypass group, was found warranted.

## 2 Materials and methods

*Overall design:* Multi center randomized controlled clinical trial.

### 2.1 Study objectives

The study was initiated to determine whether a prophylactic negative pressure wound therapy pad (PICO™) applied after vascular surgical procedures with inguinal incisions could reduce the surgical site infection rate as well as the frequency of other wound complications such, seroma/lymphocele, hematoma and wound dehiscence when compared to the standard wound dressing.

### 2.2 Endpoints

The primary endpoint is the SSI rate and secondary endpoints are the rate of seroma/lymphocele, hematoma and wound dehiscence within the first 90 days postoperatively.

### 2.3 Setting

This study is a multi-centre study including vascular centers at Skåne University Hospital, Malmö, Örebro University Hospital, Jönköping county hospital and Blekinge county hospital Karlskrona. Dedicated research nurses at each centre will keep track on inclusion, monitoring and follow up of the patients.

### 2.4 Eligibility criteria

All adult patients undergoing elective vascular procedures with incisions for arterial exposure at the lower limb are eligible to participate.

### 2.5 Enrollment and randomization

Patients scheduled for lower limb revascularization are provided with written information prior to undergoing the admission procedure which takes place one to two weeks prior to scheduled surgery. During the admission process, the background and aim of the study are discussed with eligible patients, informed consent obtained and the randomization conducted by outpatient clinic nurses. In this study we apply simple randomization using an opaque randomization envelope containing equal numbers of “PICO” and “standard” notes. In bilateral groin incisions, the draw from the envelope dictates the wound dressing selection in the right inguinal incision and the contralateral incision is automatically assigned the alternate dressing. Randomization outcome and consent form are documented in the patient's records.

### 2.6 Ethical considerations

Informed consent was obtained from all patients. The study has been registered at ClinicalTrials.gov (Identifier: NCT01913132) and approved by the regional ethical review board (Dnr 2013/322), and supplementary ethical applications were approved for the inclusion of three additional vascular centers in Örebro, Jönköping and Karlskrona (Dnr 2016/886; Decision 20161028, Dnr 2018/309; Decision 20180419, and Dnr 2019/1387; Decision 20190227, respectively).

### 2.7 The wound dressings

There is no difference between the surgical procedures and perioperative care between the treatment and control groups. At the end of the procedure, vertical incisions are closed according to surgeon's preference. Steri-Strips™ (3 M, St Paul, Minnesota) are often applied in case of intracutaneous sutures.

*PICO™* is a negative pressure system employing −80 mmHg continuous suction generated through a canister-free portable pump. It is directly applied onto the closed incisions and, according to the manufacturer's recommendation, left in place for seven days, after which it stops working and can be removed by the patients themselves or nurses at the center's outpatient clinic. Each vascular centre will use their standard wound dressing, which consists of a sterile water-proof bandage with absorbent pad.

### 2.8 Data collection and management

All data is collected using SPSS, version 25.0 (SPSS Inc., Chicago, Illinois, USA). The data stem from the patient's electronic records as well as content from conducted phone interviews after three months postoperatively. Visits at the outpatient clinic are scheduled in line with the clinic's and the Swedish vascular registry's guidelines at one and twelve months postoperatively.

#### 2.8.1 Preoperative data

Data collected preoperatively are official Swedish identification number, age at operation, date of procedure, indication for procedure, gender, height (in cm), weight (in kg), body mass index (BMI, in kg/m2), ischemic heart disease (yes/no), atrial fibrillation (yes/no), arterial hypertension (yes/no), diabetes mellitus (yes/no), type of diabetes mellitus (regulated with diet only, oral anti-diabetic drugs, insulin-dependent), current smoker (yes/no), ex-smoker (yes/no), history of cerebrovascular insult (yes/no), previous inguinal surgery on the side where the groin incision is performed (yes/no), previous extracardiac vascular surgery (yes/no), critical ischemia (yes/no), Rutherford classification for severity of lower extremity arterial disease, foot wound (yes/no), anticoagulation with warfarin (yes/no), rivaroxaban (yes/no) or dabigatran (yes/no), American Society of Anesthesiologists (ASA) score, use of systemic corticosteroids (yes/no), use of acetylsalicyclic acid (yes/no), type of procedure, groin (right/left), randomization (PICO/standard dressing).

#### 2.8.2 Perioperative data

**Fig. 1:**
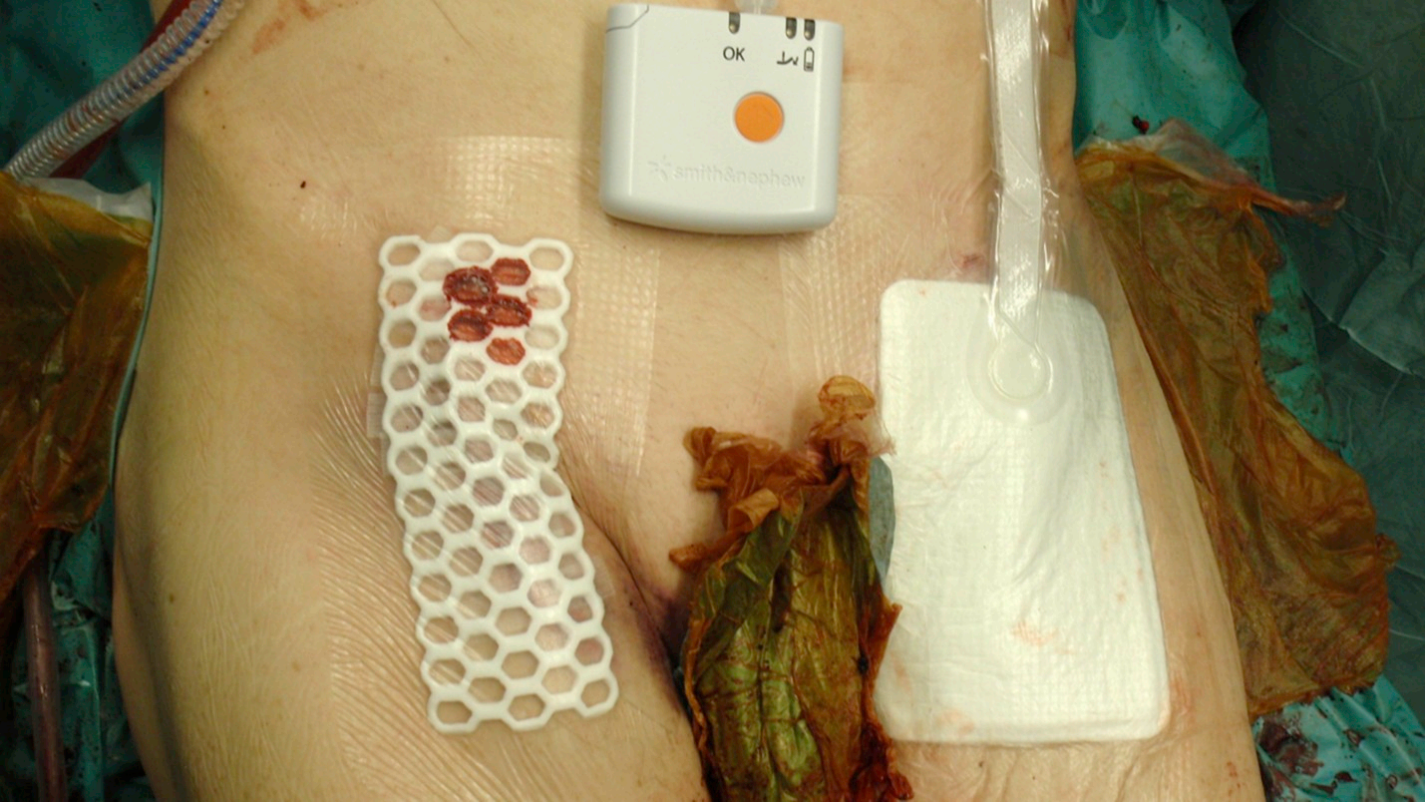
Photography of a patient who underwent bilateral thrombendarterectomy of the common and profunda femoral artery with patch angioplasty. Standard dressing (OPSITE Post-op Visible, Smith & Nephew, UK) was applied in the right groin and PICO™ (Smith & Nephew, UK) dressing in the left groin.

Preoperative antibiotic treatment excluding antibiotic prophylaxis (yes/no), preoperative antibiotic prophylaxis received (yes/no), preoperative anemia (yes/no), preoperative blood glucose concentration (mmol/L), preoperative albumin concentration (g/L), preoperative estimated glomerular filtration rate (ml/min), form of anaesthesia (general/regional/local), groin incision performed (yes/no), bilateral incision (yes/no), vertical incision (yes/no), transverse incision (yes/no), use of wound products such as Floseal® (yes/no), Hemopatch® (yes/no), Tachosil® (yes/no) (all Baxter Healthcare Corporation, Deerfield, IL, USA), Collatamp® (EUSA Pharma, Oxford, UK) (yes/no), hybrid procedure (yes/no), synthetic graft used (yes/no), bovine pericardial patch used (yes/no), any patch used (yes/no), any foreign material applied in wound (yes/no), vein graft/patch (yes/no), arterial graft (yes/no), duration of procedure (min), bypass (yes/no), wound closure technique (resorbable intracutaneous/non-resorbable mattrace sutures/staples), wound dressing received (PICO™/standard) (Fig. 1), type of standard dressing (Vitri Pad/OPSITE Post-op Visible), postoperative antibiotic therapy (yes/no), number of transfused units of packed red blood cells, postoperative treatment at intensive care unit (yes/no), inpatient length of stay (days).

#### 2.8.3 Follow-up data

Readmission within 30 days postoperatively (yes/no), surgical site infection (SSI) within 30 days (yes/no), wound culture obtained within first 3 months postoperatively (yes/no), reoperation 30 days (yes/no), sought medical attention because of groin problem within 90 days (yes/no), reoperation 90 days (yes/no), SSI 90 days (yes/no), reoperation 1 year (yes/no), SSI 1 year (yes/no), wound cultures within 1 year (yes/no), 1 year mortality (yes/no), mortality due to groin infection (yes/no), type of bacterial isolate, SSI severity grading according to Szilagyi classification [16], C-reactive protein concentration at infection diagnosis (mg/L), presenting symptoms, surgical revision (yes/no), negative pressure wound therapy after surgical revision (yes/no), sepsis (yes/no), bleeding (yes/no), wound dehiscence (yes/no), seroma (yes/no), hematoma (yes/no), lymphorrhea (yes/no), amputation within 1 year (yes/no). Adverse events of the NPWT dressing (yes/no) and type of adverse events.

The diagnosis SSI is made according to the 1999 diagnostic criteria defined by the Centers for Disease Control and Prevention, USA [17]. Wound outcome will be objectively evaluated with the validated ASEPSIS score [18]. The wound score is based upon the following criteria: Antibiotics (10 points), drainage of pus under local anaesthesia (5 points), debridement of wound (general anaesthesia) (10 points), serous discharge, erythema, purulent exudate, separation of deep tissue within the first week (5 points each), isolation of bacteria (10 points), stay as inpatient prolonged over 14 days (5 points). A total score ≥21 points is considered as infection, 11–20 points as disturbance of healing, 0–10 points as satisfactory healing. SSI along surgical incisions for exposure of arteries will be assessed. The data analysis and subsequent establishment of the diagnosis SSI or other wound complications is done under blinded conditions. The assessor at the outpatient clinic at one month and at one year does not know whether a particular groin incision was treated with NPWT or the standard wound dressing.

## 3 Analysis

The primary analysis principle applied will be intention-to-treat. The only condition for initial inclusion is application of the correct wound dressing at the end of the procedure. Exclusion criteria are early death or re-operation before being able to assess proper wound healing and thus primary or secondary endpoints. Early deaths or reoperations due to SSI are registered as SSIs.

### 3.1 Statistical issues

We defined two main groups of vascular surgical procedures with groin incisions that carry different infection risk profiles. Infection rates and other statistics stem from a review of data collected during 2014–2016 after elective open lower limb revascularization procedures at Jönköping County Hospital [15]. Reanalysis of this material was performed in order to retrieve data after elective procedure outcomes only.

In about 95% and 5% of cases, *TEA* will be performed through a unilateral respective bilateral inguinal approach. This type of procedure carries an SSI risk of 22.2%. Mortality in this group was 13.9% at one year. In about 90% and 10% of cases, bypass will be performed through a unilateral respective bilateral inguinal approach. The bypasses incorporating both groins are aorto-bifemoral or femoro-femoral bypasses. This type of procedure had an SSI rate of 41.1%. A few bypasses will not have an inguinal incision (operation for popliteal artery aneurysm). Mortality in this group was 8.9% at 1 year.

### 3.2 Sample size calculation

We used G*Power 3.1 [19] software for power calculations.

We first conducted power calculations, 80% power at 5% significance level, assuming all cases were either unilateral (Fisher's exact test, sample size n1) or bilateral (McNemar's test, sample size n2). Central to the power analysis of bilateral cases is the proportion of discordant pairs, meaning the proportion of outcomes that differ between the two sides, e.g. the proportion of cases where an infection is observed on one but not the other side. Assuming for instance a SSI rate reduction from 20% to 8% in bilateral TEA procedures, the proportion of cases in which the infection outcome is different in the right and left groin wounds respectively has to be at least as large as the difference between the current SSI rate with the standard dressing and the predicted SSI rate with the PICO™ system (20%–8% = 12%). The proportion of discordant pairs needs to be larger than the difference in SSI rate to account for the possibility that some patients may suffer from an infection on the PICO™ dressing side and not the standard dressing side.

After having determined the required sample sizes for the uni- and bilateral scenarios individually, the final sample size required was calculated as a weighted average based on the expected proportion (p) of unilateral and bilateral operations [15**]** (Table 1).

**TEA** Previous data indicated an inguinal SSI rate after TEA without bypass of 22.2% when using the standard dressing. For bilateral cases, we estimated the proportion of discordant pairs to be 20%. Assuming all cases are either unilateral or bilateral, and a reduction in SSI rate from 20% to 8%, yields n1 = 284, and n2 = 210, respectively. Among all TEA cases, 95% are assumed to be unilateral (i.e. p1 = 0.95) (Table 1):

n = p1 *n1 + (1 - p1) *n2. n = 0.95 * 284 + 0.05 * 210 = 280

**Table 1:**
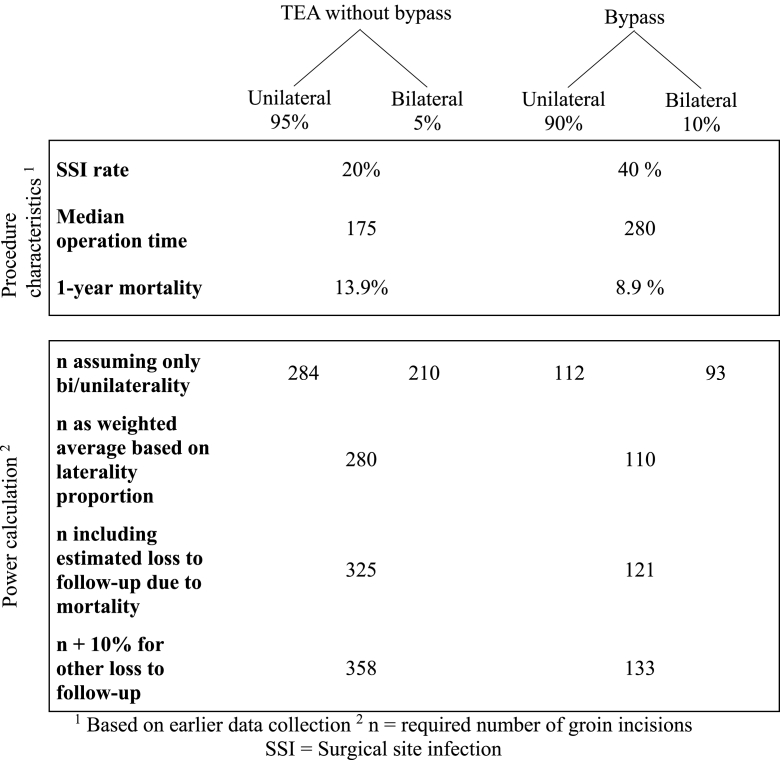
Characteristics of the two main types of procedures studied.

Taking into account the previously identified 1-year mortality of 13.9%, yields a mortality-corrected sample size of n = 325 (280/(1–0.139)).

In an attempt to adjust for other types of “loss to follow-up” such as missing data and re-operations on the respective side, we added an additional 10% (33 cases) resulting in a total sample size of 358.

**BYPASS** Previous data indicated an SSI rate after bypass of 41.1% when using the standard dressing. Assuming all cases are either unilateral or bilateral, and a reduction in SSI rate from 40% to 15% with a proportion of discordant pairs of 40% in bilateral cases yields n1 = 112 and n2 = 93, respectively. Among all bypass cases, 90% are assumed to be unilateral (i.e. p1 = 0.90) **(**Table 1**)**:

n = p1 *n1 + (1 - p1) *n2. n = 0.90 * 112 + 0.10 * 93 = 110

The 1-year mortality in this group was 8.9%, yields a mortality-corrected sample size of n = 121 (110/(1–0.089)). To adjust for other types of “loss to follow-up”,we added an additional 10% (12 cases) resulting in a total sample size of 133.

## 4 Statistical analysis

We will conduct a descriptive analysis of background data such as comorbidities, procedure-related risk factors and different operation techniques. Important differences will be accounted for in a sensitivity analysis.

The study involves both uni- and bilateral inguinal incisions. Unilateral incisions are treated with either standard or NPWT dressing according to the randomization result and analyzed with *Fisher's exact test for independent samples*. In case of bilateral incisions, one is randomly designated to either standard or NPWT treatment group, the other by default to the alternate treatment group. It is important to recognize that the outcomes in the treatment groups in this scenario will not be independent of one another and will therefore be analyzed with *McNemar's test for paired data*. The advantage of the bilateral design is that all patient-related risk factors, such as comorbidities and hygiene factors, are exactly the same in both treatment groups and patients serve as their own control. The bilateral design thus decreases the required group size at a fixed level of statistical power, as can be seen from the assessments of n1 and n2 above. The obtained p-values from the uni- and bilateral analyses will subsequently be combined to an overall p-value using *Fisher's method of combining p-values* [20]. The e logarithm of the two p-values are calculated, summed, and multiplied with 2, resulting in a chi-square distribution with four degrees of freedom and a corresponding combined p-value (Excel sheet calculation). By using *McNemar's test* for analysis of bilateral incisions, we avoid the problem of “clustering” due to statistical dependent observations which if not accounted for would lead to too small standard errors and p-values [21].

## 5 Discussion

Vascular surgery with inguinal incisions carries a significant risk for SSI. Extensive need for incisions in the lower limb increases the risk of SSI. Incisional NPWT could be an important tool in the postoperative care of these anatomically and microbiologically challenging wounds. A central effect of incisional NPWT in inguinal or other lower limb incisions could simply be to provide a high-quality protective cover to the healing wound for a seven-day period. The constant suction that is being applied by the incisional NPWT-system provides a highly-adaptive surface that prevents kinking of the wound edges and a subsequent breach of the sterile barrier resulting in bacterial contamination during movements of the hip joint.

Only elective procedures will be included. This is primarily due to ethical concerns in including patients in need of an emergency operation. Previous surveys using a validated wound infection register found a 4% SSI rate after emergency operation and 27% SSI rate after elective vascular procedures [22]. This much higher SSI rate after elective vascular surgery has been speculated to be a consequence of poor patient environment, hygiene facilities and routines at the ward for vascular surgery patients prior to surgery.

A potential limitation of this study could be that the randomization takes place before the operation, not at its end. One might argue that this could create bias since the surgeon in charge, could be aware of the respective randomization outcome before conducting the operation and thereby subconsciously or consciously modify their behavior and surgical technique. Because of the large staff turn-over in the operation room, an intraoperative randomization would most likely have led to a very large number of patient dropouts due to insufficient sense of awareness for the study and the principal investigator felt that intraoperative computer-generated randomization not seems feasible. Use of randomization envelopes in the operation room was not felt as an option either due to issues of management of these envelopes and the large turnover of personnel at present times. When wound closure has been performed, the surgeon usually leaves the operation room and the scrub nurse finalizes the wound care including application of wound dressings. That is usually the time to take a look at the randomization results in the patient files, which should minimize bias due to behavior issues. At present time, four vascular centers will be enrolling patients into the study and these centers does not, for instance, have identical perioperative wound care. The type of standard wound dressing used in the control arm might differ. We will, however, document the type of wound dressing and perform sub analysis accordingly. Generalizability of the results will be better in a multi-centre trial including both university hospitals and county hospitals.

Prospective RCT studies are prone to the Hawthorne effect, leading to changes in behavior among personnel and patients [23]. This may affect the results in both the interventional and control arms of this study. To address this issue a parallel qualitative study was initiated in Jönköping. The aim of this randomized controlled trial is to provide more high-quality evidence as to the effects of incisional NPWT after vascular surgery with groin and lower limb incisions, in particular TEA and lower limb bypass. To avoid bias, the authors have decided to publish the rationale and methods of this study, especially the sample size calculation, prior to enrollment completion and analysis. The results of the trial could not only be important for postoperative wound management in vascular patients, but could be relevant to other specialties conducting operations with incisions at high risk of SSI.

## Funding sources

SA was supported by grants from Research Funds at Skåne University Hospital, Region Skåne, the Hulda Ahlmroth Foundation, and from the Swedish Government under the ALF agreement.

## Declaration of competing interest

None.
